# Distinct Suppression of Prednisone on Endometrial Immune Cells in Women With Reproductive Failure

**DOI:** 10.1111/aji.70151

**Published:** 2025-10-07

**Authors:** Lu Chen, Xiaoyan Chen, Qianhan Xu, Joshua Jing Xi Li, Wen‐Jui Yang, Hanbin Wu, Chi Chiu Wang, Xiaodan Fan, Jie Ji, Jacqueline Pui Wah Chung, Mingqing Li, Tin Chiu Li, Tao Zhang

**Affiliations:** ^1^ Faculty of Medicine Department of Obstetrics and Gynaecology The Chinese University of Hong Kong Hong Kong China; ^2^ Department of Obstetrics and Gynaecology Maternal Fetal Medicine Institute Shenzhen Baoan Women's and Children's Hospital Jinan University Shenzhen China; ^3^ Department of Pathology School of Clinical Medicine The University of Hong Kong Queen Mary Hospital Hong Kong China; ^4^ Department of Infertility and Reproductive Medicine Taiwan IVF Group Center Hsinchu Taiwan; ^5^ Reproduction and Development Laboratory Li Ka Shing Institute of Health Sciences The Chinese University of Hong Kong Hong Kong China; ^6^ School of Biomedical Sciences The Chinese University of Hong Kong Prince of Wales Hospital Hong Kong China; ^7^ Chinese University of Hong Kong—Sichuan University Joint Laboratory in Reproductive Medicine The Chinese University of Hong Kong Hong Kong China; ^8^ Department of Statistics The Chinese University of Hong Kong Hong Kong China; ^9^ Department of Reproductive Immunology School of Medicine The International Peace Maternity and Child Health Hospital Shanghai Jiao Tong University Shanghai China

**Keywords:** endometrium, macrophages, NK cells, prednisone, reproductive failure

## Abstract

**Research Question:**

For women with reproductive failure, prednisone is widely used but remains controversial as a therapy for improving clinical outcomes. This study aimed to investigate the impact of prednisone on various endometrial immune cells.

**Design:**

A total of 24 women with repeated implantation failure and recurrent miscarriage underwent their first endometrial biopsy precisely 7 days after luteinizing hormone surge (LH surge +7). Prednisone was administered from the first day of the subsequent menstrual cycle, followed by the second endometrial sampling on day LH surge +7 again. The density and cell–cell clustering of CD3−CD56+CD16− NK cells, CD3−CD56+CD16+ NK cells, CD68+CD16− macrophages, CD68+CD16+ macrophages, CD3+CD56− T cells, and CD3+CD56+ NK‐T cells were analyzed by multiplex staining and compared before and after prednisone treatment.

**Results:**

Following prednisone treatment, a significant decrease in the percentage of CD3−CD56+CD16− NK cells (*p <* 0.001), CD68+CD16− macrophages (*p* = 0.007), and their clustering degree (*p* = 0.038) was observed. No significant changes were noted in CD3−CD56+CD16+ NK cells, CD68+CD16+ macrophages, CD3+CD56− T cells, CD3+CD56+ NK‐T cells, and their cell–cell clustering.

**Conclusion:**

Prednisone does not generally reduce all endometrial immune cells; instead, it selectively suppresses specific immune cell subtypes in women with reproductive failure.

## 1 Introduction

Endometrial immune cells undergo dynamic adaptations in preparation for various biological events during production. Specifically, during the window of implantation (WOI) and early pregnancy, there is a robust increase in uterine natural killer (NK), followed by macrophages [1]. These immune cells play an active role in facilitating decidualization, spiral artery remodeling, and trophoblast invasion, thereby maintaining a supportive and compatible microenvironment essential for successful embryo implantation and placentation [2].

The disruption of endometrial immune cells during the WOI has been linked to reproductive failure, including recurrent miscarriage (RM) [1] and repeated implantation failure (RIF) [3]. Among the different types of endometrial immune cells, NK cells have been the subject of extensive studies. These cells are typically characterized by the presence of CD56+CD16− makers in the majority and CD56+CD16+ markers in the minority [4, 5]. CD16 (FcγRIII) is an IgG receptor involved in antibody‐dependent cell cytotoxicity, enhances NK cell cytotoxic potential, and supports cytokine production, thereby promoting the immune response against foreign, infected, or malignant cells. Generally, CD56+CD16+ NK cells are considered a cytotoxic phenotype, while CD56+CD16− NK cells are a regulatory phenotype due to their high production of growth factors and chemokines. A recent meta‐analysis of 44 studies revealed a significant increase in total CD56+ endometrial NK cells during the WOI in women with reproductive failure compared to fertile controls [6]. Interestingly, when subgroup analysis was conducted based on the markers used to identify NK cells, no significant differences were observed in endometrial CD56+CD16− NK cells between women with RM and controls [6]. This suggests that the main increase in total NK cells in RM may be attributed to the CD56+CD16+ cytotoxic subpopulation. Unfortunately, this subgroup analysis cannot be conducted in women with RIF due to limited studies utilizing two markers to identify NK cells. In contrast to the extensive research on NK cells, only a few studies have indicated a substantial increase in inflammatory macrophages, cytotoxic T cells, and NK‐like T cells in women with reproductive failure [7, 8, 9]. Considering these remarkable changes, endometrial immune cells have emerged as potential therapeutic targets for improving pregnancy outcomes in women with reproductive failure [10, 11].

Prednisone is a corticosteroid used to suppress an overactive immune response and has been widely studied for its potential to enhance embryo implantation and prevent miscarriage [12, 13, 14, 15, 16]. A recent large‐scale randomized clinical trial has shown that prednisone does not improve the live birth rate in women with RIF when compared with a placebo [17]. Despite ongoing debates regarding its therapeutic benefits, prednisone was administered based on clinical diagnosis rather than the status of endometrial immune status in most studies [17, 18]. Therefore, investigating the impact of prednisone on endometrial immune cells could provide valuable insights into identifying suitable indications and enhancing the therapeutic effects of prednisone in future clinical trials.

Previous studies have demonstrated the suppressive effects of prednisone on total CD56+ NK cells [19, 20]. However, using CD56 alone to identify NK cell phenotypes is inadequate, as CD56 is also expressed by NK‐T cells, a heterogeneous group exhibiting characteristics of both T and NK cells [21]. Furthermore, CD56 cannot distinguish between CD56+CD16− NK cells and CD56+CD16+ NK cells. As a result, our current understanding of the effect of prednisone on endometrial immune cells remains limited, hindering the identification of appropriate indications for its use.

This study aims to compare various endometrial immune cells, including CD3−CD56+CD16− and CD3−CD56+CD16+ NK cells, CD68+CD16− and CD68+CD16+ macrophages, CD3+CD56− T cells, and CD3+CD56+ NK‐T cells before and after prednisone treatment. Additionally, their spatial clustering patterns are compared as emerging evidence indicate that spatial proximity between immune cells may reflect not only structural arrangement but also functional interactions [22, 23, 24].

## 2 Materials and Methods

### 2.1 Subjects

Women with reproductive failure, including those with RM or RIF, were recruited for this study from 2019 to 2023. All procedures were conducted in accordance with the Declaration of Helsinki and obtained ethical approval from the Joint Chinese University of Hong Kong‐New Territories East Cluster Clinical Research Ethics Committee (CREC Ref: 2018.586; 2023.056). Specifically, 13 patients were recruited from Prince of Wales Hospital, and 11 patients were recruited from the Union Hospital. The informed form was waived for the use of unstained slides obtained from archived samples in the Department of Pathology, Prince of Wales Hospital. Informed consent was obtained from the participant before the study if a pathological test was not performed. RM was defined as two or more miscarriages before 24 weeks of gestation [25], while RIF was defined as the failure to achieve a clinical pregnancy after transferring at least four good‐quality embryos over a minimum of three fresh or frozen cycles [26]. The inclusion criteria were as follows: (a) nonsmokers; (b) aged 20–42 years; (c) regular menstrual cycles (25–35 days); (d) elevated endometrial NK cells, defined as above the normal reference range (1.2%∼4.5%), which was established based on the fifth and 95th percentiles of CD56+NK cells in 72 fertile controls [27]. The exclusion criteria included the following: (a) presence of hydrosalpinx; (b) structural uterine abnormalities, identified through three‐dimensional ultrasonography, such as fibroid, endometrial polyp, or intrauterine adhesions; (c) parental chromosomal abnormalities; (d) Day‐2 follicle‐stimulating hormone (FSH) >10 IU/L or mid‐luteal progesterone <30 nmol/L; (e) significant medical conditions such as systemic lupus erythematosus, which are known to affect the immune system; (f) abnormal thyroid function; and (g) intake of any antibiotics, estrogen or progestogen hormonal therapy, steroid treatment, or intrauterine contraceptive device within 3 months prior to recruitment. A total of 153 women with reproductive failure were recruited to measure endometrial NK cells. Among them, 29 patients were identified as having elevated levels of endometrial NK cells. Of these, five patients withdrew from the study for personal reasons, resulting in 24 women included in the final analysis.

### 2.2 Endometrial Biopsy

Participants underwent daily urine dipstick tests from Day 9 of the menstrual cycle to detect the LH surge, indicating ovulation. This surge was used to precisely time the endometrial biopsies on the seventh day post‐LH surge during the peri‐implantation phase. Biopsies were obtained by utilizing either a Pipelle sampler (Prodimed, Neuilly‐en‐Thelle, France) or a Pipet Curet (Cooper Surgical, Trumbull, CT). The specimens were promptly fixed in 10% neutral buffered formalin at room temperature overnight, followed by dehydration in a series of ethanol solutions and embedding in paraffin wax.

#### 2.2.1 Administration of Prednisone

In the menstrual cycle following the initial biopsy, oral prednisone (10 mg/day) was administered from menstrual Days 1–3 until LH surge +7, when the second biopsy was performed. This dosage was selected based on prior clinical trials demonstrating effective immune modulation and safety in reproductive failure populations [19, 28]. The regimen was then tapered: 5 mg/day for 3 days, 2 mg/day for 3 days, and 1 mg/day for 3 days.

### 2.3 Multiplex Immunohistochemical Staining Workflow

Multiplex immunohistochemical staining (m‐IHC) with tyramide signal amplification (TSA) was performed using the Opal 7‐color Manual IHC kit (Akoya Biosciences), as previously described [29]. Briefly, formalin‐fixed paraffin‐embedded sections underwent sequential staining rounds for CD3 (1:100, ab16669; Abcam), CD16 (1:100, ab183354; Abcam), CD68 (1:100, ab192847; Abcam), and CD56 (1:100, 504; Leica). Each round included primary antibody incubation, HRP‐conjugated secondary antibody (1x Anti‐Ms + Rb HRP, 10 min), and TSA signal amplification with Opal fluorophores (620, 570, 520, 690; diluted 1:100 in amplification diluent). Between rounds, HRP was inactivated by microwave heating in AR6 buffer (2‐min boiling, 15‐min low power). Nuclei were counterstained with DAPI (1:10, Akoya Biosciences), and slides were mounted with ProLong Antifade (Thermo). Full protocol details are provided in Supporting Materials (Figure S1A,B).

### 2.4 Cell Counting Methodology

**FIGURE 1:**
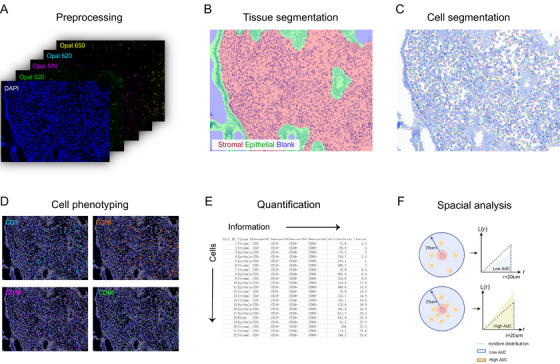
Analysis flowchart for using Mantra and Inform software. Upon capturing the image using the Mantra workstation, the initial step involves preprocessing the image to separate various fluorescence channels (A). Subsequently, through manual training, the software autonomously identifies epithelium and stroma in the endometrium (B) and segments each individual cell (C). It further classifies cells based on the expression level of markers on the cell surface (D). Once all analyses are completed, relevant information for each cell is extracted (E) and imported into the R language to determine the degree of aggregation among different cell types (F).

A systematic approach was employed for cell counting to ensure accuracy and consistency. A minimum of five fields were captured at 200× magnification using the Mantra Quantitative Pathology Workstation, guaranteeing a minimum of 10 000 stromal cells per sample. The first field captured was selected at random, ensuring that it contained the luminal epithelial border. Subsequent fields were obtained by moving either to the left or right of the original field while maintaining the visibility of the luminal epithelial border. If there were insufficient fields containing epithelial cells, the field below the selected ones was included. This meticulous methodology in selecting the sampling area is crucial to prevent the inclusion of NK cell aggregates, which tend to be located deeper within the tissue [27, 30]. Following image acquisition, the collected images were processed through advanced supervised machine‐learning algorithms (Inform software, version 2.4.6, PerkinElmer) to segment each region of interest, delineating epithelial and stromal compartments (Figure 1). Subsequently, sophisticated supervised machine‐learning techniques, utilizing the built‐in functionalities of Inform software, were employed to classify various immune cells. This classification relied on a combination of immune cell marker expression profiles and cellular morphology. This refined approach enabled the accurate identification of distinct immune cell phenotypes, including CD3−CD56+CD16− NK cells, CD3−CD56+CD16+ NK cells, CD68+CD16− macrophages, CD68+CD16+ macrophages, CD3+CD56− T cells, and CD3+CD56+ NK‐T cells (Figure S1C). The quantified cell‐level data were then expressed as a percentage relative to the total number of stromal cells present in each image. In addition, the proportions of NK cell and macrophage subsets within their respective parent populations (i.e., CD3−CD56+ cells and CD68+ cells) were also calculated to assess changes in their phenotypic composition.

### 2.5 Intraobserver and Interobserver Variability

To assess intraobserver variability, cell counting was performed by one observer using the Inform Cell Analysis system on 10 randomly selected slides. The counting was repeated on two separate occasions, with the observer blinded to the results of the previous measurements.

Interobserver variability was evaluated by two independent observers who analyzed the same set of 10 randomly chosen slides. Each observer counted the cells according to the established protocol, ensuring independent assessments.

### 2.6 Quantification of Endometrial Immune Cell Spatial Distribution

We used the Inform software for tissue and cell segmentation and spatial distribution analysis, methods previously validated in our study on patients with reproductive failure [31]. The *X* and *Y* coordinates of each cell were considered as a bivariate point pattern and examined under a microscope at 200× magnification [32]. Bivariate K‐ and L‐functions, derived from Ripley's K‐ and L‐functions, were employed to describe the spatial configuration. The L‐function estimation was performed utilizing the “spatstat” package in R, over a range of *r* values from 0 to 20 µm [32]. The degree of clustering among different pairs of immune cells was determined by calculating the area under the curve (AUC) of their respective L‐functions using the R program. A higher AUC value indicated a greater degree of cell–cell clustering, reflecting an increased likelihood of interactions, while a lower AUC value denoted a lower degree of clustering (Figure 1).

### 2.7 Statistical Analysis

The normality of data distribution was assessed using the Shapiro–Wilk test. The paired *t*‐test was used for normally distributed data, assuming equal variance, and the Wilcoxon signed‐rank test for non‐normally distributed data to assess treatment‐related changes within the same patient over time. Both tests were two‐sided. The Spearman correlation coefficient was calculated for two quantitative parameters and displayed in a scatter diagram. The relationship between baseline immune cell proportions and their treatment‐related changes was also assessed by Spearman correlation. Statistical significance was set at *p* < 0.05. All statistical analyses were performed with IBM SPSS Statistics (version 26, 2019).

## 3 Results

### 3.1 Demographics

In this study, a cohort of 24 participants was recruited to compare cell density and clustering of various endometrial immune cells before and after prednisone treatment. The demographic characteristics of all patients are detailed in Table 1.

**TABLE 1:** Demographics of women included in the study.

	All (*n* = 24)
Age (years)	37.7 ± 3.5
Body mass index (kg/m^2^)	22.6 ± 2.1
Gravida	2 (0–5)
Parity	0 (0–1)
FSH (IU/L)	7.1 ± 1.5

### 3.2 Staining Patterns

**FIGURE 2:**
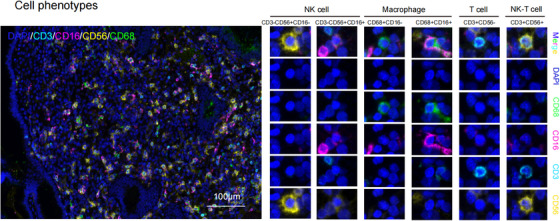
Phenotyping of endometrial immune cells. The staining panel identifies two phenotypes of NK cells (CD3−CD56+CD16− and CD3−CD56+CD16+), two macrophage phenotypes (CD68+CD16− and CD68+CD16+), as well as CD3+CD56+ NK‐T cells and CD3+CD56− T cells can be identified by the staining panel. Scale bar  =  100 µm.

Figure 2 shows a representative image of m‐IHC staining for CD3−CD56+CD16− NK cells, CD3−CD56+CD16+ NK cells, CD68+CD16− macrophages, CD68+CD16+ macrophages, CD3+CD56− T cells, and CD3+CD56+ NK‐T cells.

### 3.3 Intraobserver and Interobserver Variability

**FIGURE 3:**
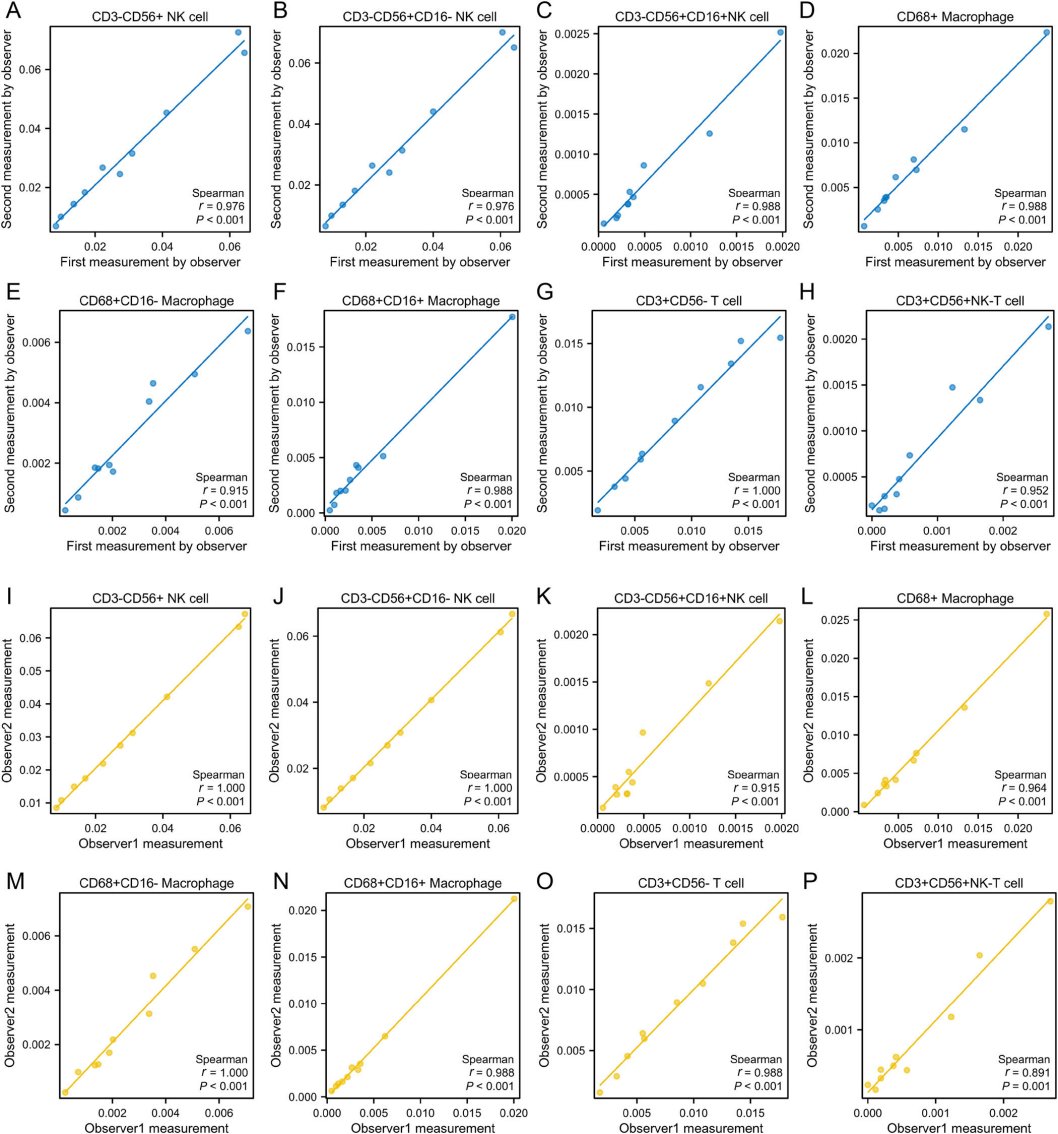
Intra‐ and interobserver variability in immune cell density measurements in the endometrium. The scatter plots demonstrate the correlation between measurements taken by a single observer on two separate occasions (A–H) and measurements taken by two independent observers (I–P). *r*, Spearman correlation.

To evaluate both intraobserver and interobserver variability in measuring the density of various immune cell types, 10 slides were selected for repeated measurements by the same observer and by two independent observers. As shown in Figure 3, significant correlations were observed for intraobserver measurements: CD3−CD56+ NK cells (*r* = 0.976, *p* < 0.001), CD3−CD56+CD16− NK cells (*r* = 0.976, *p* < 0.001), CD3−CD56+CD16+ NK cells (*r* = 0.988, *p* < 0.001), CD68+ macrophages (*r* = 0.988, *p* < 0.001), CD68+CD16− macrophages (*r* = 0.915, *p* < 0.001), CD68+CD16+ macrophages (*r* = 0.0.988, *p* < 0.001), CD3+CD56− T cells (*r* = 1.000, *p* < 0.001), and CD3+CD56+ NK‐T cells (*r* = 0.952, *p* < 0.001).

Furthermore, significant correlations were also found for interobserver measurements: CD3−CD56+ NK cells (*r* = 1.000, *p* < 0.001), CD3−CD56+CD16− NK cells (*r* = 1.000, *p* < 0.001), CD3−CD56+CD16+ NK cells (*r* = 0.915, *p* < 0.001), CD68+ macrophages (*r* = 0.964, *p* < 0.001), CD68+CD16− macrophages (*r* = 1.000, *p* < 0.001), CD68+CD16+ macrophages (*r* = 0.988, *p* < 0.001), CD3+CD56− T cells (*r* = 0.988, *p* < 0.001), and CD3+CD56+ NK‐T cells (*r* = 0.891, *p* < 0.001) between the measurements of observers A and B.

### 3.4 Absolute Changes in Immune Cell Densities Before and After Prednisone Treatment

#### 3.4.1 NK Cells

**FIGURE 4:**
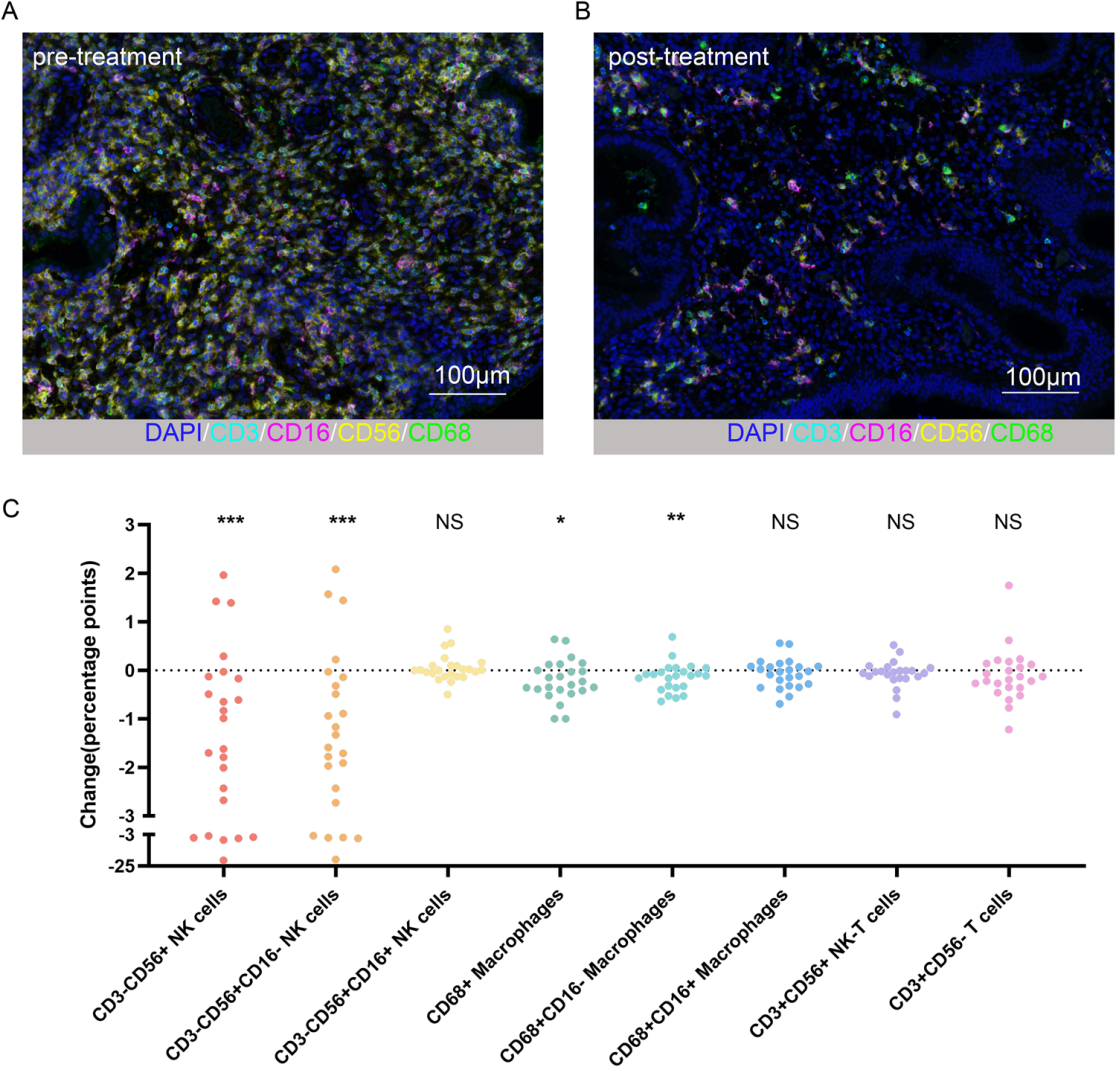
Representative images and absolute changes in endometrial immune cells before and after prednisone treatment. Representative images show the changes in endometrial immune cells before and after prednisone treatment (A, B), indicating a decrease in cell density. Figure (C) illustrates the absolute changes (in percentage points) of various immune cell types post‐treatment, with paired comparisons revealing significant reductions in CD3−CD56+ NK cells, CD3−CD56+CD16− NK cells, CD68+ macrophages, and CD68+CD16− macrophages (*p < 0.05; **p ≤ 0.01; ***p ≤ 0.001). Changes were calculated as the difference between post‐treatment and pretreatment immune cell densities (scale bar = 100 µm). Paired *t*‐tests were used for normally distributed data, while Wilcoxon signed‐rank tests were applied to non‐normally distributed data.

Following prednisone treatment, a significant decrease in the percentage of CD3−CD56+ NK cells was observed in women with reproductive failure (*p <* 0.001) (Figure 4). The percentage of CD3−CD56+ NK cells decreased from a median of 6.18% (range: 4.50%–24.29%) before treatment to 4.18% (range: 1.05%–8.72%) after treatment. Notably, the percentage of CD3−CD56+CD16− NK cells decreased significantly compared to baseline (*p <* 0.001), moving from a median of 6.12% (range: 4.11%–23.63%) before treatment to 4.00% (range: 1.02%–8.61%) after treatment. In contrast, no significant difference was observed in the change in the percentage of CD3−CD56+CD16+ NK cells, with the median percentage changing from 0.11% (range: 0.00%‐0.72%) before treatment to 0.12% (range: 0.00%–0.96%) after treatment (Table 2).

**TABLE 2:** Comparison of density for different immune cells pre‐ and post‐treatment.

Cell type	Pretreatment cell density (%), median (range)	Post‐treatment cell density (%), median (range)	Median rate of decrease after treatment	*p* value
CD3−CD56+ NK cells	6.18 (4.50–24.29)	4.18 (1.05–8.72)	25.00% (−31.11–86.29)	<0.001
CD3−CD56+CD16− NK cells	6.12 (4.11–23.63)	4.00 (1.02–8.61)	27.17% (−33.76–86.60)	<0.001
CD3−CD56+CD16+ NK cells	0.11 (0.00–0.72)	0.12 (0.00–0.96)	1.82% (−535.4–100.00)	0.747
CD68+ macrophages	0.74 (0.10–2.15)	0.68 (0.00–2.15)	28.96% (−606.60–99.14)	0.02
CD68+CD16− macrophages	0.62 (0.07–1.46)	0.27 (0.00–1.70)	15.95% (−81.23–98.43)	0.007
CD68+CD16+ macrophages	0.24 (0.02–1.01)	0.20 (0.00–0.82)	16.38% (−1256.00–99.49)	0.293
CD3+CD56+ NK‐T cells	0.15 (0.02–1.28)	0.09 (0.01–0.83)	38.64% (−1064.00–91.20)	0.115
CD3+CD56− T cells	0.87 (0.17–2.67)	0.86 (0.25–2.31)	17.26% (−592.10–62.28)	0.084

*Note:* The normality of data distribution was assessed using the Shapiro–Wilk test. For normally distributed data, a paired *t*‐test was employed, assuming equal variance, while the Wilcoxon signed‐rank test was used for non‐normally distributed data to evaluate treatment‐related changes within the same patient over time.

#### 3.4.2 Macrophages

Following prednisone administration, the percentage of CD68+ macrophages significantly decreased compared to the pretreatment baseline (*p* = 0.02), from a median of 0.74% (range: 0.10%–2.15%) before treatment to 0.68% after treatment (range: 0.00%–2.15%). Further categorization based on the co‐expression of CD16 delineated CD68+CD16+ macrophages and CD68+CD16− macrophages. The percentage of CD68+CD16− macrophages significantly declined after treatment compared to baseline (*p* = 0.007), moving from a median of 0.62% (range: 0.07%–1.46%) before treatment to 0.27% after treatment (range: 0.00%–1.70%). In contrast, no significant difference was observed in the change in the percentage of CD68+CD16+ macrophages, with the median percentage changing from 0.24% (range: 0.02%–1.01%) before treatment to 0.20% after treatment (range: 0.00%–0.82%) (Table 2).

#### 3.4.3 T Cells and NK‐T Cells

The administration of prednisone did not result in any notable alterations in the percentages of CD3+CD56− T cells (*p* = 0.084, with the median changing from 0.87% (range: 0.17%–2.67%) before treatment to 0.86% after treatment (range: 0.25%–2.31%). Similarly, for CD3+CD56+ NK‐T cells, no significant difference was observed (*p* = 0.115), with the median changing from 0.15% (range: 0.02%–1.28%) before treatment to 0.09% after treatment (range: 0.01%–0.83%) (Table 2).

### 3.5 Relationship Between Baseline Proportions and Absolute Changes in NK Cells and Macrophages Following Prednisone Administration

To further investigate whether the significant reduction in NK cells and macrophages following prednisone administration was associated with their baseline proportions, we performed a Spearman correlation analysis. The baseline proportions of CD3−CD56+ total NK cells and CD3−CD56+CD16− NK cells did not show a significant correlation with their corresponding reductions following prednisone administration (*r* = 0.360, *p* = 0.084; *r* = 0.330, *p* = 0.116, respectively). Similarly, no significant correlations were found between the baseline levels of CD68+ macrophages or CD68+CD16− macrophages and their respective reductions (*r* = 0.214, *p* = 0.345; *r* = 0.102, *p* = 0.636, respectively).

### 3.6 Changes in Immune Cell Subset Composition Following Prednisone Treatment

The proportions of NK cell and macrophage subsets within their respective parent populations were compared before and after prednisone treatment (Table S1). The proportion of CD3−CD56+CD16− NK cells among total CD3−CD56+ NK cells decreased significantly from 98.09% (range: 91.61%–100.00%) to 96.94% (range: 73.70%–100.00%) after treatment (*p* = 0.011). In contrast, the proportion of CD3−CD56+CD16+ NK cells increased from 1.91% (range: 0.00%–8.39%) to 3.06% (0.00%–26.30%).

The proportion of CD68+CD16− macrophages among total CD68+ macrophages decreased from 67.84% (range: 24.26%–97.96%) to 55.01% (range: 17.88%–90.34%) after treatment, although the change was not statistically significant (*p* = 0.225). Correspondingly, CD68+CD16− macrophages increased from 32.16% (range: 2.04%–75.54%) to 44.99% (range: 9.66%–82.12%).

### 3.7 Alterations in Immune Cell Aggregation Levels Before and After Prednisone Treatment

**FIGURE 5:**
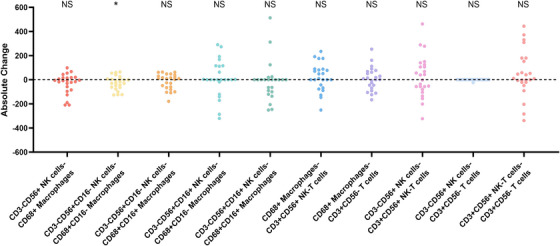
Absolute changes in clustering between immune cells before and after prednisone treatment. The absolute changes in the clustering between distinct pairs of endometrial immune cells are shown. Paired comparisons reveal a significant reduction in clustering between CD3−CD56+CD16− NK cells and CD68+CD16− macrophages following prednisone treatment. The paired *t*‐tests were used for normally distributed data, while the Wilcoxon signed‐rank test was used for non‐normally distributed data. *p < 0.05.

Figure 5 illustrates the changes in clustering between the two types of immune cells. Following prednisone treatment, no significant difference was observed in the clustering of CD3−CD56+ NK cells and CD68+ macrophages compared to baseline in women with reproductive failure (*p* = 0.082), with a median changing from 190.40 (range: 96.52–328.70) before treatment to 176.90 after treatment (range: 0.00–250.30). However, further analysis revealed a significant reduction in clustering between CD3−CD56+CD16− NK cells and CD68+CD16− macrophages (*p* = 0.038), moving from a median of 189.30 (range: 24.68–280.20) before treatment to 155.10 after treatment (range: 0.00–305.90). Notably, no significant differences were found in clustering changes of CD3−CD56+CD16− NK cells with CD68+CD16+ macrophages, CD3−CD56+CD16+ NK cells with CD68+CD16− macrophages, or CD68+CD16+ macrophages. Additionally, clustering levels among other cell types remained unaltered before and after treatment (Table 3).

**TABLE 3:** Alterations in the clustering patterns of distinct pairs of endometrial immune cells before and after treatment in all patients with reproductive failure.

Immune Cell Pairs	Pretreatment AUC Median (range)	Post‐treatment AUC Median (range)	*p* value
CD3+CD56+ NK cells vs. CD68+ macrophages	190.40 (96.52–328.70)	176.90 (0.00–250.30)	0.082
CD3+CD56+CD16− NK cells vs. CD68+CD16− macrophages	189.30 (24.68–280.20)	155.10 (0.00–305.90)	0.038
CD3+CD56+CD16‐ NK cells vs. CD68+CD16+ macrophages	167.10 (0.00–211.60)	102.80 (0.00–220.80)	0.486
CD3+CD56+CD16+ NK cells vs. CD68+CD16− macrophages	0.00 (0.00–320.60)	27.44 (0.00–298.70)	0.734
CD3+CD56+CD16+ NK cells vs. CD68+CD16+ macrophages	88.72 (0.00–291.10)	0.00 (0.00–513.1)	0.391
CD68+ macrophages vs. CD3+CD56+ NK‐T cells	51.18 (0.00–323.20)	76.28 (0.00–275.20)	0.352
CD68+ macrophages vs. CD3+CD56− T cells	168.80 (6.45–296.40)	175.10 (0.00–260.20)	0.806
CD3+CD56+ NK cells vs. CD3+CD56+ NK‐T cells	301.10 (0.00–426.30)	318.90 (0.00–477.30)	0.638
CD3+CD56+ NK cells vs. CD3+CD56− T cells	212.20 (103.80–412.00)	227.80 (75.12–354.40)	0.486
CD3+CD56+ NK‐T cells vs. CD3+CD56− T cells	214.40 (0.00–581.70)	243.0 (0.00–649.90)	0.231

*Note:* The normality of data distribution was assessed using the Shapiro–Wilk test. For normally distributed data, a paired *t*‐test was employed, assuming equal variance, while the Wilcoxon signed‐rank test was used for non‐normally distributed data to evaluate treatment‐related changes within the same patient over time.

## 4 Discussion

Despite the lack of available tests to evaluate endometrial immune status and diagnose endometrial receptivity, prednisone is commonly used to suppress the inflammatory response in the endometrium, aiming to enhance embryo implantation and prevent miscarriage [33, 34]. However, the effects of prednisone on endometrial immune cells remain limited. Our study is the first to demonstrate that prednisone administration significantly reduces the percentage of CD3−CD56+CD16− NK cells and CD68+CD16− macrophages, as well as their clustering degree. In contrast, there are no significant changes in other immune cell densities and clustering after prednisone treatment. These notable changes in specific immune cell types suggest that prednisone selectively suppresses certain immune cell phenotypes rather than affecting all phenotypes.

### 4.1 Decrease in CD56+CD16− NK Cells

Under physiological conditions, endometrial NK cells play regulatory roles in facilitating angiogenesis to prepare the endometrium for embryo implantation. However, women with reproductive failure exhibit a significant increase in endometrial NK cells, which has been correlated with elevated vascular density. This suggests that excessive accumulation of NK cells may produce an overabundance of pro‐angiogenic cytokines, promoting excessive angiogenesis that could potentially lead to oxidative stress and, subsequently, reproductive failure [35, 36, 37]. Therefore, an excessive number of endometrial NK cells might not be beneficial for endometrial preparation, despite their critical regulatory roles. Additionally, our recent study found that, in addition to CD56+CD16− regulatory NK cells, CD56+CD16+ cytotoxic NK cells were also significantly increased during the WOI in women with reproductive failure [4]. This increase was also observed in the decidua of women with RM [38]. Although several immunosuppressive agents have been shown to reduce total NK cell numbers [19, 20], it remains unclear whether both the regulator and cytotoxic subsets can be equally decreased.

In the present study, we observed a significant reduction in the percentage of CD3−CD56+ NK cells following prednisone treatment in women with reproductive failure and elevated NK cell percentage, which is consistent with previous findings [19]. Co‐localization staining with CD16 revealed that this reduction was primarily due to a significant decrease in the CD3−CD56+CD16− regulatory NK cell subset, rather than a decrease in the CD3−CD56+CD16+ cytotoxic NK cells. The decrease in CD3−CD56+CD16− NK might help restore the disrupted local environment caused by excessive accumulation of regulatory NK cells. However, it must be carefully monitored to avoid over‐inhibition, considering its crucial roles in endometrial preparation for embryo implantation and placentation [39]. More importantly, the reduction in the proportion CD56+CD16− NK cells within the total NK cell population led to a relative increase in the proportion of CD56+CD16+ NK cells, suggesting a phenotypic shift toward cytotoxicity. This shift has been correlated with implantation failure and early miscarriage [38, 40]. The insufficient suppression of the CD16+NK subset by prednisone may help explain the lack of positive results regarding its efficacy in improving the live birth rate and reducing miscarriage rate in women with reproductive failure [19, 41, 42]. These findings suggest that prednisone may only be beneficial for women with elevated CD56+CD16− NK cells, rather than for those with increases in both NK subsets. Hence, it is critical to establish a normal reference range for each NK cell subset to identify appropriate candidates for treatment.

The mechanisms underlying the different responses to prednisone between CD3−CD56+CD16− NK cell subset and CD3−CD56+CD16+ NK cells remain unclear. While it has been reported that NK cells can express the glucocorticoid (GC) receptor, the expression levels have not been assessed in the context of reproductive failure or within NK cell subsets [43]. Further investigation is necessary to elucidate this potential avenue.

### 4.2 Decrease in CD68+CD16− Macrophages

The percentage of endometrial macrophages doubles during the secretory phase compared to the proliferative phase, making them the second most abundant immune cells during WOI [44, 45]. Macrophages are generally classified based on surface markers and functions into either “classically activated” macrophages (M1) or “alternatively activated” macrophages (M2), which lead to pro‐ or anti‐inflammatory responses, respectively, depending on the local microenvironment [46, 47]. Endometrial macrophages typically exhibit an M2‐like phenotype and contribute to angiogenesis, tissue repair, and anti‐inflammatory processes by producing various cytokines and growth factors [48, 49]. A study by Quenby et al. found that women who had miscarriages following endometrial biopsy had significantly more macrophages compared to those who subsequently had a live birth [50]. A more recent study has indicated that CD163+ M2‐like endometrial macrophages can predict clinical pregnancy in women undergoing in vitro fertilization–embryo transfer (IVF‐ET) [51]. Taken together, poor reproductive and pregnancy outcomes may be associated with an increase in M1‐like endometrial macrophages and a decrease in M2‐like macrophages.

In our study, co‐localization of CD68 and CD16 was utilized to identify M1‐like macrophages, which showed no significant change after prednisone treatment. However, the percentage of CD68+CD16− macrophages, including M2 and unclassified subsets, was significantly reduced. Given that M2 macrophages play critical roles in preparing the endometrium for embryo implantation [52, 53, 54], the suppression of CD68+CD16− macrophages by prednisone could impair their physiological functions during endometrial preparation. This, in turn, caused a relative increase in CD68+CD16+ M1‐like macrophages within the total CD68+ macrophage population, although such an increase was not statistically significant. These findings suggest a slight shift towards M1 polarization, potentially compromising reproductive and pregnancy outcomes.

Similar to NK cells, endometrial macrophages can express GR, but the expression levels across different subsets and subsequently mediated functions in these macrophages remain largely unexplored [55, 56]. Further studies are required to elucidate why different macrophage subsets respond differently to prednisone.

### 4.3 Decrease in NK Cell–Macrophage Clustering

Emerging evidence suggests that decidual NK cells interact with macrophages to facilitate angiogenesis and placentation [57, 58], as well as maintain an immunotolerant microenvironment through decreasing NK cell cytotoxicity [59], promoting M2 polarization [60], and inducing differentiation of T regulatory cells (Tregs) [61]. However, the specifics of this cell–cell interaction during the WOI remain unexplored due to the challenge of isolating sufficient cell numbers from fresh samples for in vitro co‐culture studies. Advances in multiplex immunofluorescence staining offer a promising avenue to assess cell–cell interactions based on the spatial distribution. Recent studies analyzing spatial proximity between NK cells and macrophages in tuberculosis granuloma have shown that shorter cell–cell distance can reflect their interactions, such as antigen presentation, and cytotoxicity pathways [22]. Our previous study analyzed the clustering between CD56+ NK cells and CD68+ macrophages and revealed a significant increase in women with RM compared to controls, suggesting their increased interaction is correlated with RM [31].

In the present study, we observed a notable reduction in the distance between CD56+CD16− NK cells and CD68+CD16− macrophages, but not other subtypes, following prednisone treatment in women with reproductive failure. This suggests that prednisone not only reduces the density of these cells but also diminishes their interactions. However, it remains unclear whether this suppression can improve IVF‐ET outcomes, given the crucial roles of these interactions in embryo implantation and placentation. Further studies are needed to investigate the specific immune cell interactions involved in reproductive failure before this issue can be fully addressed.

### 4.4 Unchanged Parameters

Unlike NK cells and macrophages, endometrial T cells significantly decrease in proportion from being the predominant immune cells, accounting for 40%∼60% of total immune cells during the proliferative phase, to only 10% during the luteal phase [62]. Various types of T cells, including memory T cells, regulatory T cells, NK‐T cells, cytotoxic T cells, and T helper (Th) cells, have been identified in the endometrium, involved in local immune response and tolerance [7, 63]. Previous studies have shown that increased CD3+ T cells and NK‐T cells during the WOI are associated with reproductive failure [7, 63]. The present study showed that prednisone did not significantly affect the percentage of CD3+CD56− T cells and CD3+CD56+ NK‐like T cells. The nonresponsiveness of T cells and NK‐like T cells to prednisone treatment may be attributed to selective responses and varying expression levels of GR. While immune cells generally express GR [64], their response to GCs can be selective. Previous studies have shown that pro‐inflammatory T cell subsets, such as Th1 and Th17 cells, exhibit a significantly higher responsiveness to GCs compared to Th2 cells [65]. However, since the various subsets of T cells were not investigated in the present study, we are unable to determine which T cell subset is responsive to prednisone. Additionally, in certain pathological conditions, such as chronic inflammation, the expression of GR may be downregulated in some NK‐likeT cells, potentially reducing their responsiveness to GC‐mediated suppression [66]. The endometrium of women with RM and RIF has been shown to be characterized by an inflammatory microenvironment [67, 68]. Whether this accounts for the observed differential responsiveness warrants further investigation.

In addition to interacting with NK cells, macrophages also engage with T cells. A recent study using single‐cell RNA sequencing found that the interaction between macrophages and T cells in the decidua was notably increased in women with RM compared to controls. Moreover, decidual macrophages in women with RM are prone to interact with Th1‐like T cells [69]. The spatial distance between T cells and antigen‐presenting cells has also been analyzed to reflect T cell–mediated immune pathways and predict prognosis in cancers [24]. Subsequently, our previous study investigated the cell–cell distance between macrophages and T cells during WOI and found it was not significantly changed in women with RM compared to fertile controls [29]; however, the clustering with different T cell subtypes was not determined. In the present study, the integration between CD68+CD16+ or CD68+CD16− macrophages and T cells was not significantly changed after the administration of prednisone, suggesting prednisone may not affect their interaction. Nevertheless, identifying T cell subtypes and increasing the sample size are necessary to confirm this conclusion.

### 4.5 Strengths and Limitations

This study employed a multiplex staining method, which proved to be a particular strength, allowing for precise identification of specific immune cells and their subtypes by analyzing the co‐localization of two to three markers. This approach overcomes the limitations of the conventional immunohistochemical (IHC) method, which can only broadly identify one immune cell type based on a single marker. Another advantage of multiplex staining over traditional IHC staining is its ability to automatically and rapidly measure a large number of cells (>10 000 cells), effectively excluding nonspecific and background signals while eliminating subjective bias. Additionally, this technique facilitates the analysis of spatial relationships between different cell types, providing insights into the impact of prednisone on cell–cell interaction.

A further strength of this study is its design, which compares the changing patterns of immune cells within the same subjects before and after prednisone treatment. By allowing each subject to act as her own control, this design effectively reduces variance and enhances the reliability of the results compared to a traditional cohort study. Moreover, to account for the dramatic changes in NK cell density during the mid‐luteal phase, endometrial samples were precisely collected on Day 7 after the LH surge, both before and after the administration of prednisone. This careful timing mitigates the potential influence of biopsy timing on endometrial immune cells.

However, our study has certain limitations. The endometrial immune cell profile between RIF and RM may not be exactly the same, although we recruited patients with higher levels of uNK cells [27]. Subgroup analysis within each specific population was not feasible due to the small sample size. Future studies with larger cohorts are warranted to explore potential subgroup differences. Additionally, although we analyzed cell–cell clustering to reflect their interaction, the spatial clustering does not necessarily equate to functional enhancement. Its pathophysiological significance should be further evaluated through cytokine profiling, signaling pathway analysis, and functional studies. Another limitation of our study is that we recruited women with elevated CD56+ NK cells based on the normal reference range we previously established. However, due to the lack of a normal reference range for each NK subset, we are unable to determine which subset was elevated the most. One more limitation is that we did not explore their correlation with IVF‐ET outcomes. This omission means we cannot assess the potential impact of these immune cell changes on clinical outcomes, such as implantation rates and miscarriage rates following prednisone treatment. Future studies with larger sample sizes will be necessary to investigate the relationship between immune cell dynamics and reproductive success.

## 5 Conclusion

Prednisone specifically suppresses CD3−CD56+CD16− NK cells and CD68+CD16− macrophages, along with their interactions, in women with reproductive failure. The clinical implications of these specific changes in immune cells following prednisone treatments warrant further investigation.

## Ethics Statement

Ethical approval was obtained from our institutional review board at the Joint Chinese University of Hong Kong‐New Territories East Cluster Clinical Research Ethics Committee (CREC Ref: 2018.586; 2023.056).

## Conflicts of Interest

The authors declare no conflicts of interest.

## Supporting information




**Table S1**: Proportional changes in NK and macrophage subsets pre‐ and post‐treatment

**Figure S1**: Analysis flowchart for using Mantra and Inform software

## Data Availability

The data that support the findings of this study are available from the corresponding author upon reasonable request.
