# Supplementary material for: Distinct Suppression of Prednisone on Endometrial Immune Cells in Women With Reproductive Failure
Source: Am J Reprod Immunol. 2025 Oct 7;94(4):e70151. doi: 10.1111/aji.70151 (PMC12503086; doi:10.1111/aji.70151)
Supplement: Supplementary file 1 — Table S1: Proportional changes in NK and macrophage subsets pre‐ and post‐treatment [file AJI-94-e70151-s001.pptx]

## Slide 1
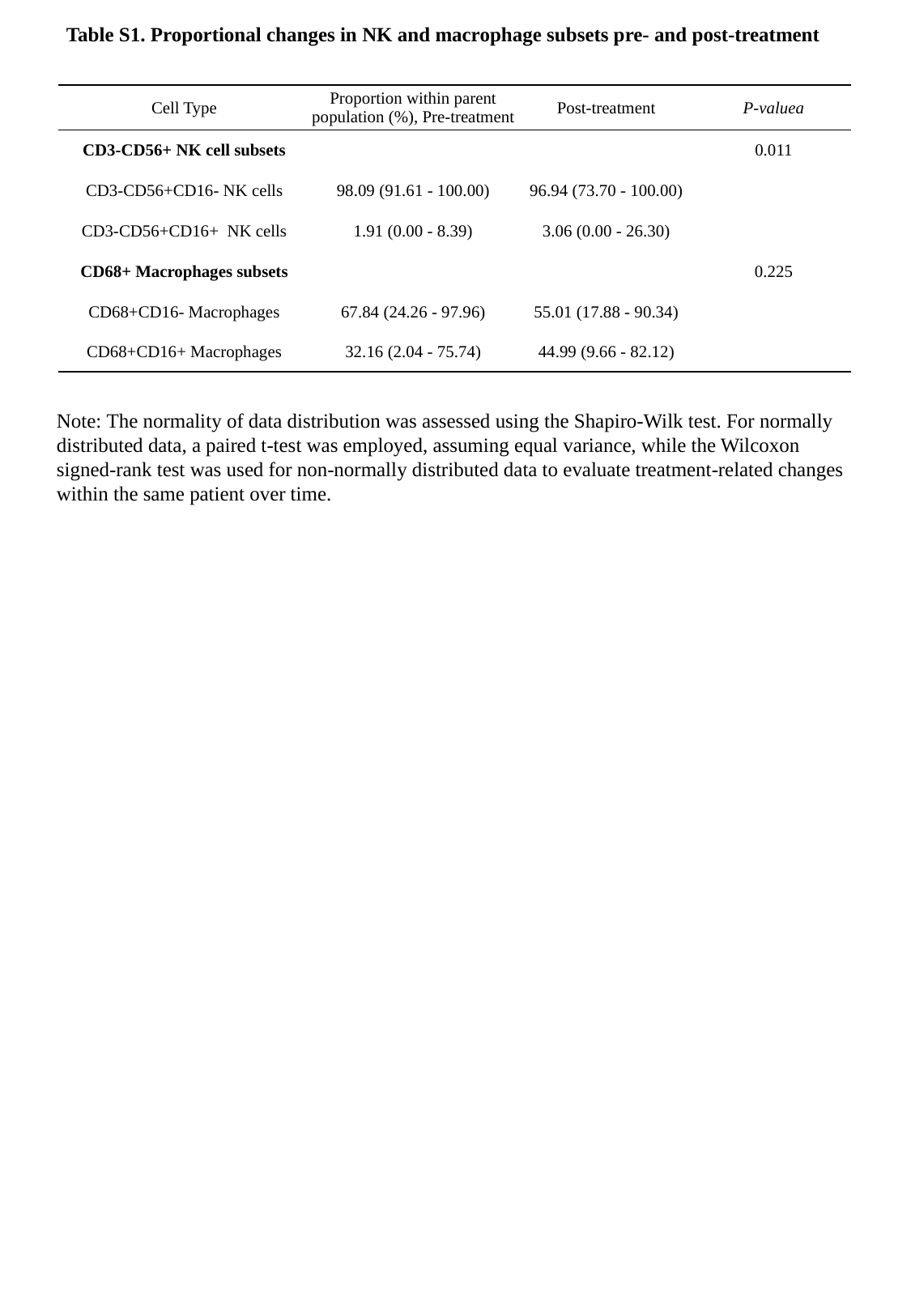

Table S1. Proportional changes in NK and macrophage subsets pre- and post-treatment
| Cell Type | Proportion within parent population (%), Pre-treatment | Post-treatment | P-valuea |
| --- | --- | --- | --- |
| CD3-CD56+ NK cell subsets | | | 0.011 |
| CD3-CD56+CD16- NK cells | 98.09 (91.61 - 100.00) | 96.94 (73.70 - 100.00) | |
| CD3-CD56+CD16+ NK cells | 1.91 (0.00 - 8.39) | 3.06 (0.00 - 26.30) | |
| CD68+ Macrophages subsets | | | 0.225 |
| CD68+CD16- Macrophages | 67.84 (24.26 - 97.96) | 55.01 (17.88 - 90.34) | |
| CD68+CD16+ Macrophages | 32.16 (2.04 - 75.74) | 44.99 (9.66 - 82.12) | |
Note: The normality of data distribution was assessed using the Shapiro-Wilk test. For normally distributed data, a paired t-test was employed, assuming equal variance, while the Wilcoxon signed-rank test was used for non-normally distributed data to evaluate treatment-related changes within the same patient over time.
