# Supplementary material for: Distinct Suppression of Prednisone on Endometrial Immune Cells in Women With Reproductive Failure
Source: Am J Reprod Immunol. 2025 Oct 7;94(4):e70151. doi: 10.1111/aji.70151 (PMC12503086; doi:10.1111/aji.70151)
Supplement: Supplementary file 2 — Figure S1: Analysis flowchart for using Mantra and Inform software [file AJI-94-e70151-s002.pdf]

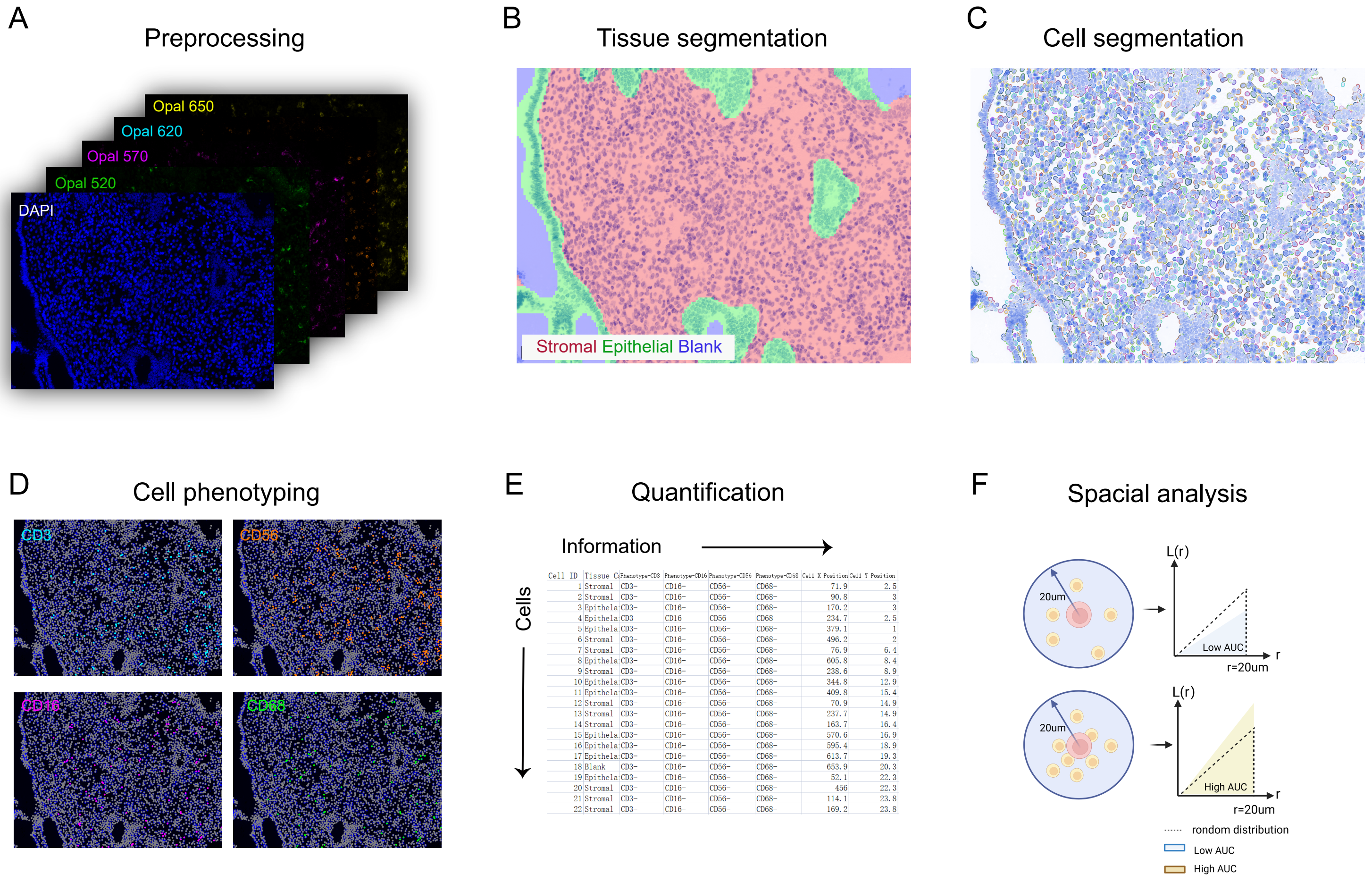

Cell ID	Tissue Ca	Phenotype-CD3	Phenotype-CD16	Phenotype-CD56	Phenotype-CD68	Cell X Position	Cell Y Position
1	Stromal	CD3-	CD16-	CD56-	CD68-	71.9	2.5
2	Stromal	CD3-	CD16-	CD56-	CD68-	90.8	3
3	Epithela:	CD3-	CD16-	CD56-	CD68-	170.2	3
4	Epithela:	CD3-	CD16-	CD56-	CD68-	234.7	2.5
5	Epithela:	CD3-	CD16-	CD56-	CD68-	379.1	1
6	Stromal	CD3-	CD16-	CD56-	CD68-	496.2	2
7	Stromal	CD3-	CD16-	CD56-	CD68-	76.9	6.4
8	Epithela:	CD3-	CD16-	CD56-	CD68-	605.8	8.4
9	Stromal	CD3-	CD16-	CD56-	CD68-	238.6	8.9
10	Epithela:	CD3-	CD16-	CD56-	CD68-	344.8	12.9
11	Epithela:	CD3-	CD16-	CD56-	CD68-	409.8	15.4
12	Stromal	CD3-	CD16-	CD56-	CD68-	70.9	14.9
13	Stromal	CD3-	CD16-	CD56-	CD68-	237.7	14.9
14	Stromal	CD3-	CD16-	CD56-	CD68-	163.7	16.4
15	Epithela:	CD3-	CD16-	CD56-	CD68-	570.6	16.9
16	Epithela:	CD3-	CD16-	CD56-	CD68-	595.4	18.9
17	Epithela:	CD3-	CD16-	CD56-	CD68-	613.7	19.3
18	Blank	CD3-	CD16-	CD56-	CD68-	653.9	20.3
19	Epithela:	CD3-	CD16-	CD56-	CD68-	52.1	22.3
20	Stromal	CD3-	CD16-	CD56-	CD68-	456	22.3
21	Stromal	CD3-	CD16-	CD56-	CD68-	114.1	23.8
22	Stromal	CD3-	CD16-	CD56-	CD68-	169.2	23.8

Figure 1. Analysis flowchart for using Mantra and Inform software.

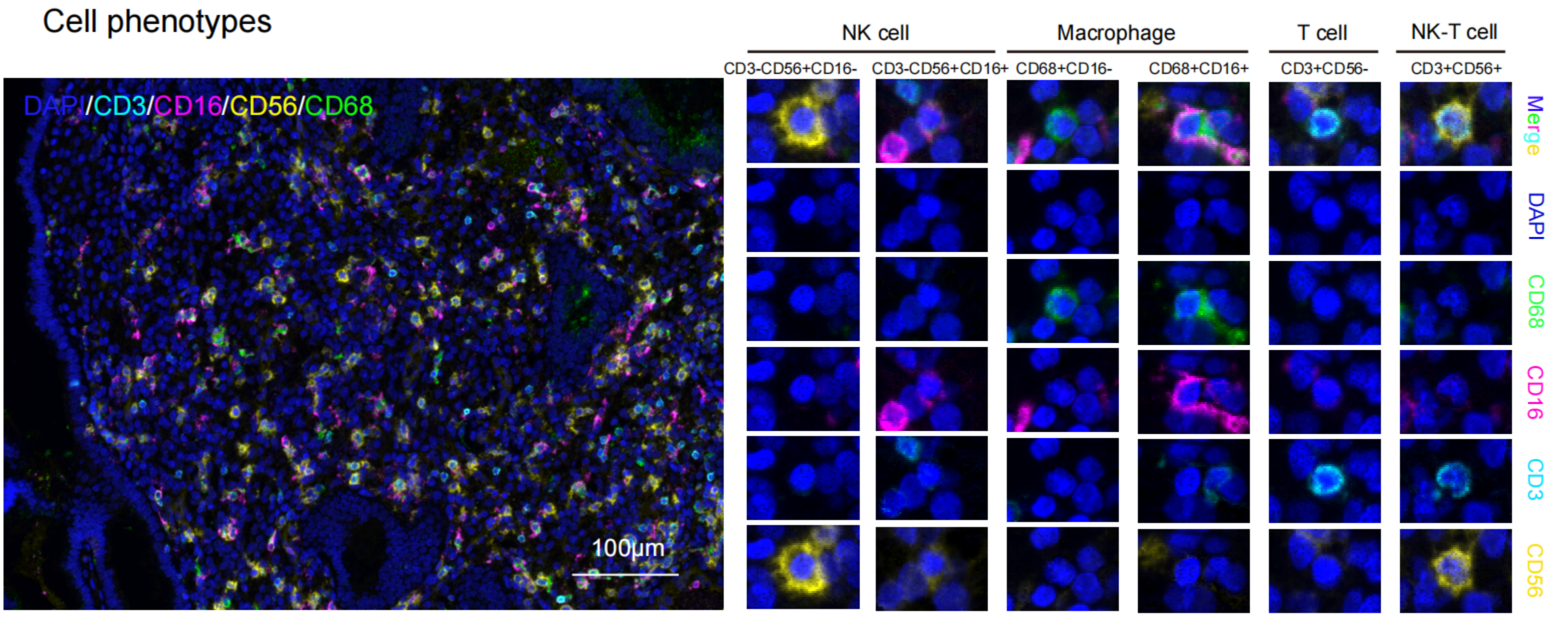

Figure 2. Phenotyping of endometrial immune cells

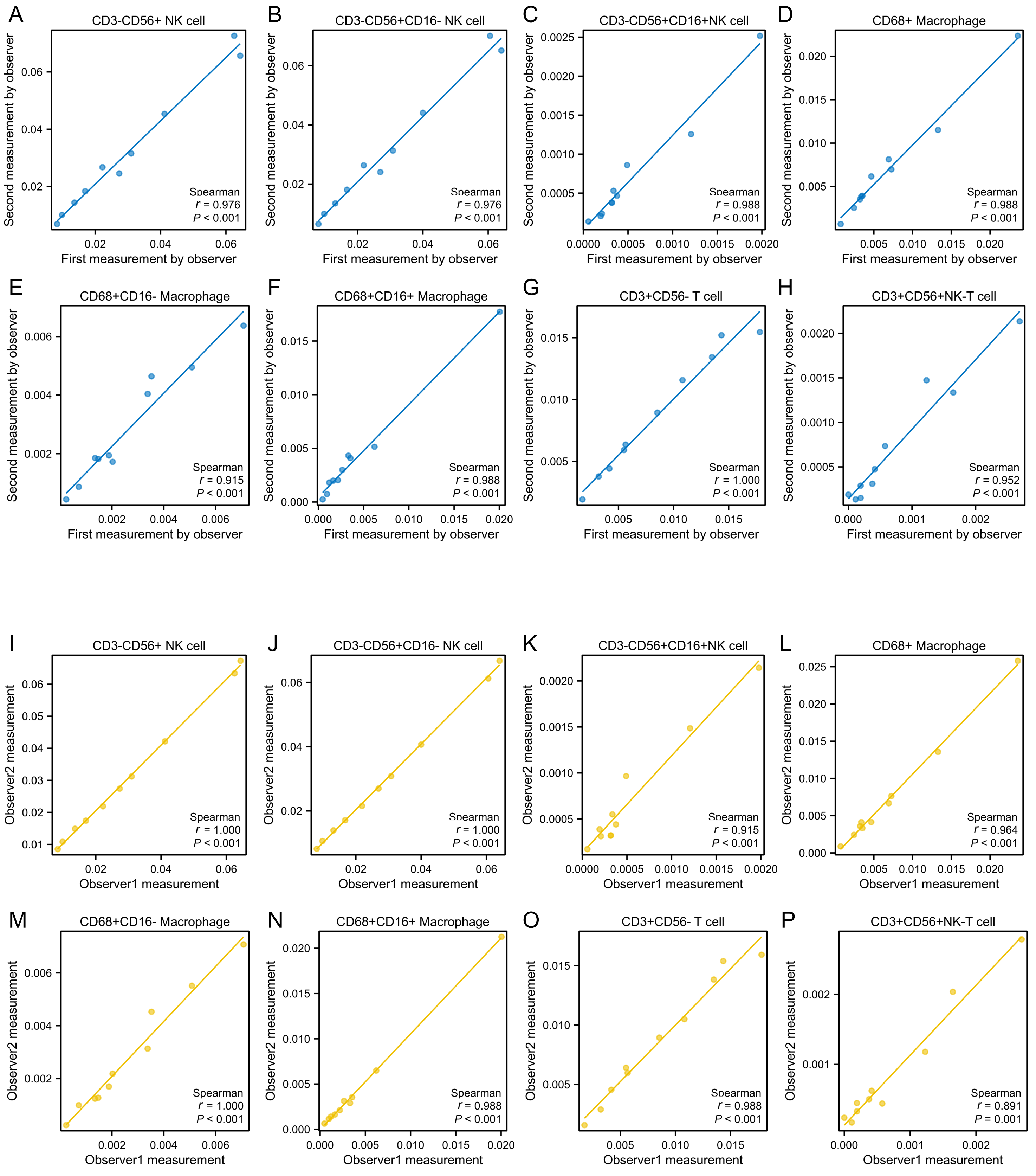

Figure 3. Intra- and interobserver variability in immune cell density measurements in the endometrium.

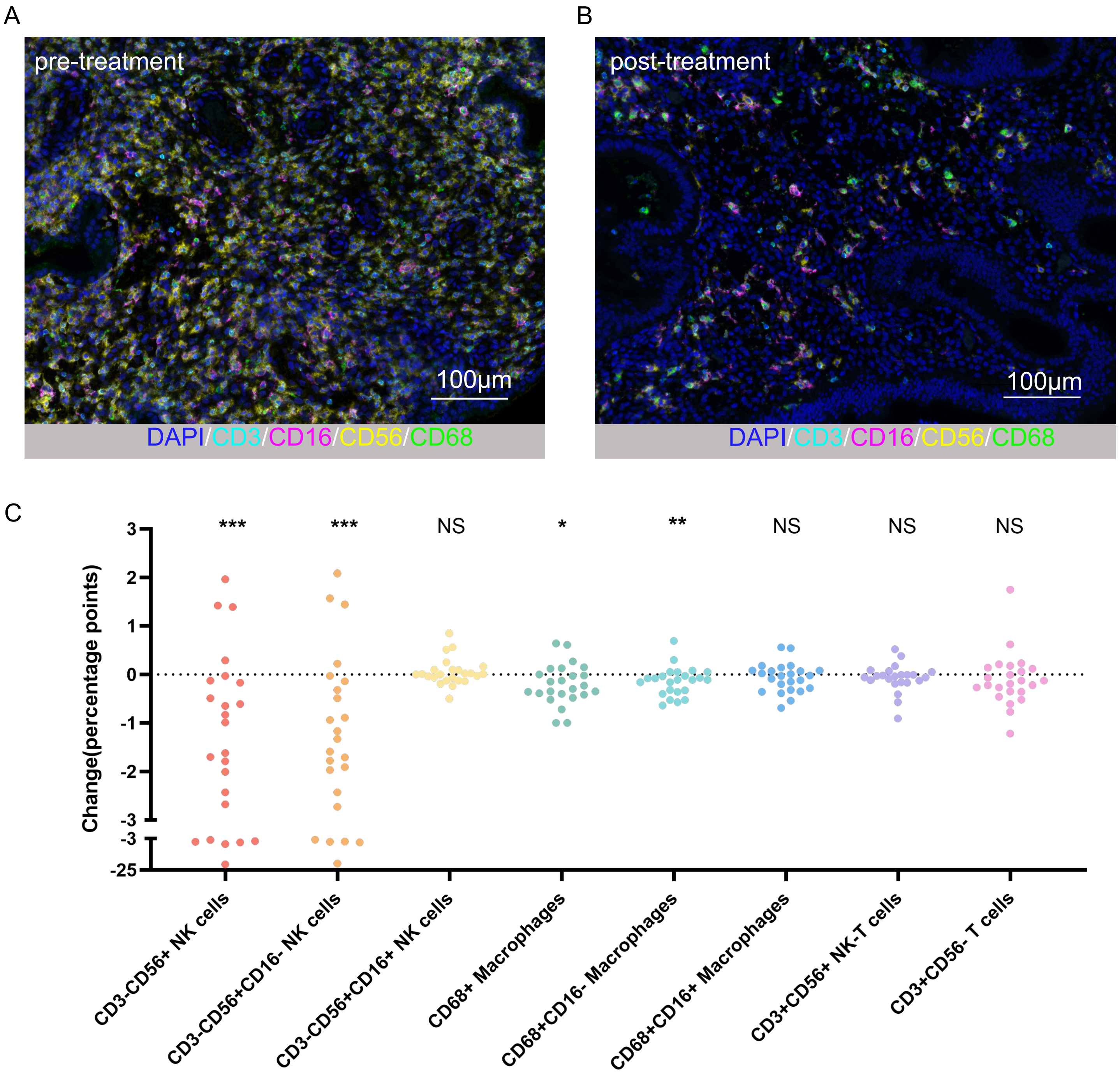

Figure 4. Representative images and absolute changes in endometrial immune cells before and after prednisone treatment.

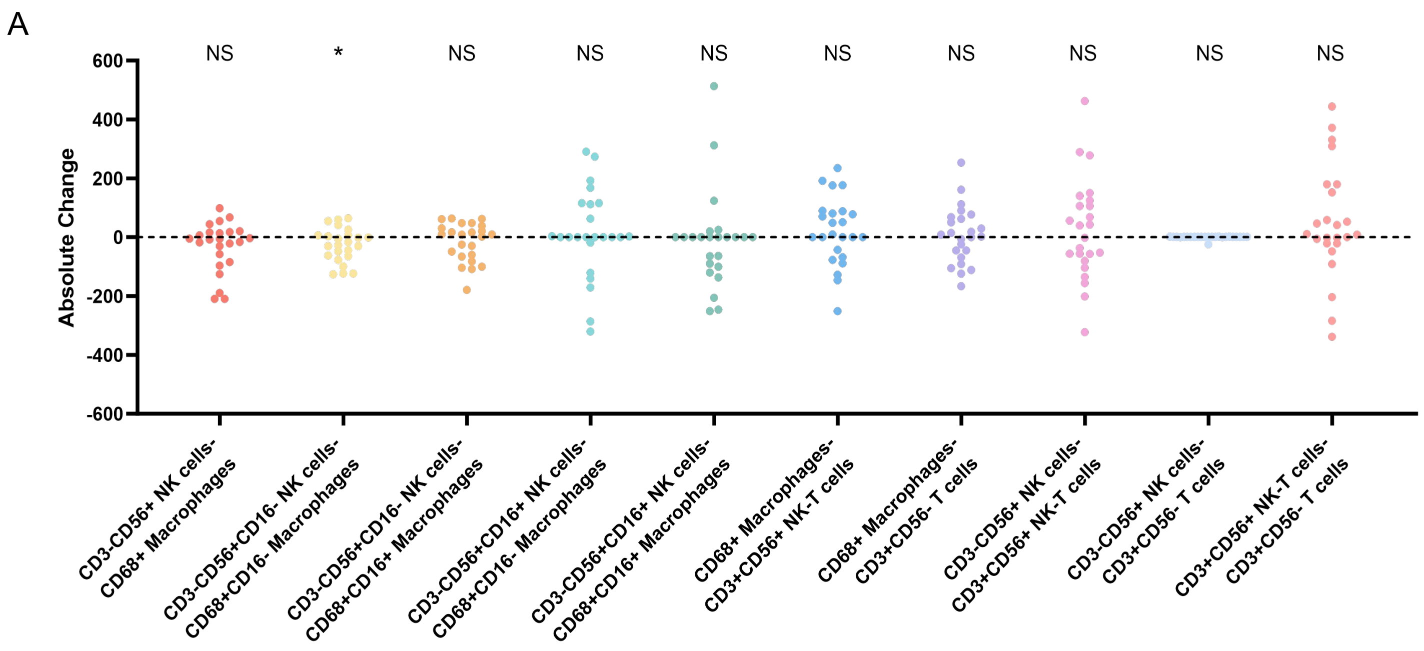

Figure 5. Absolute changes in clustering between immune cells before and after prednisone treatment.

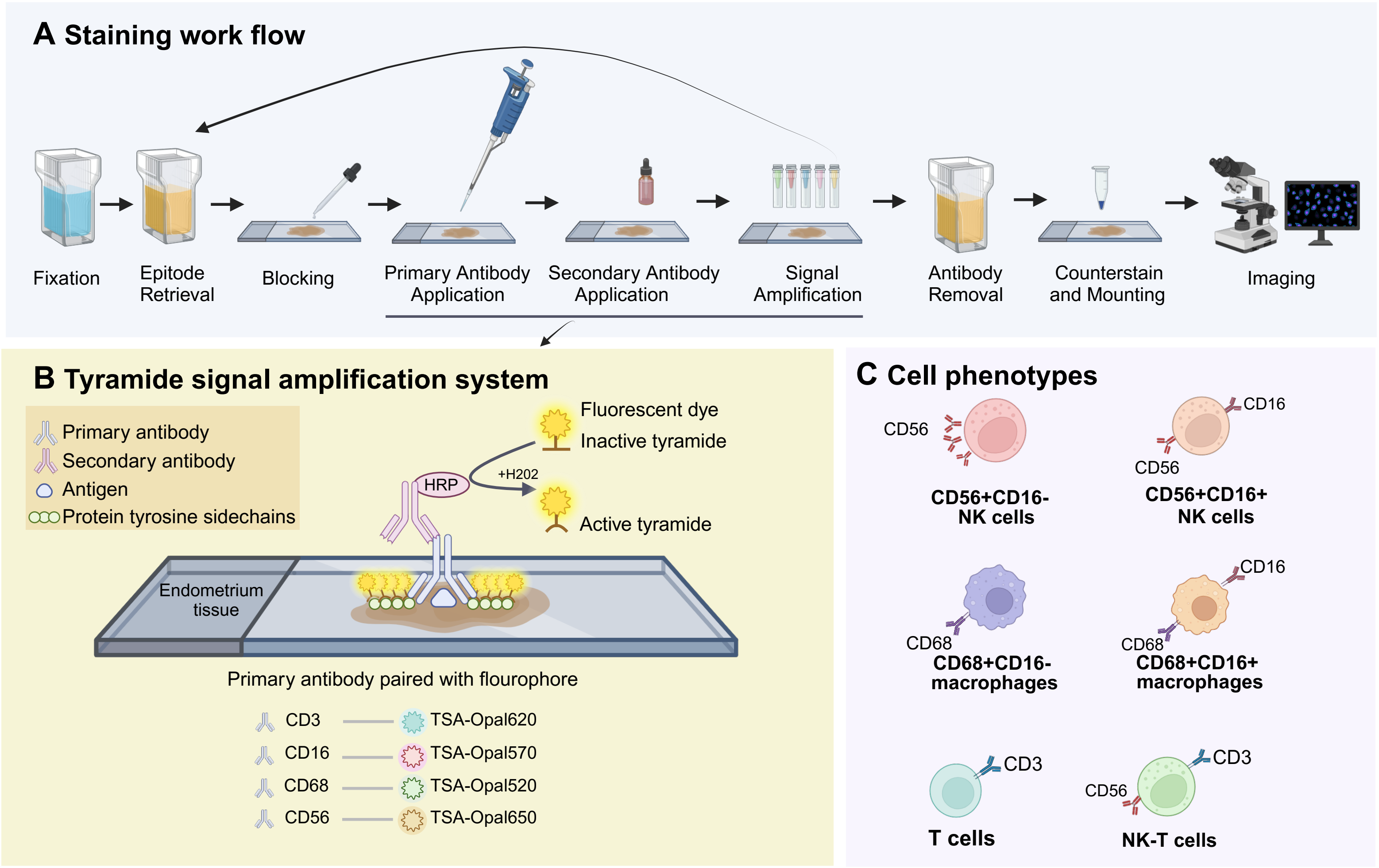

Supplementary Figure 1. Methodology and identification of endometrial immune cell subtypes through m-IHC staining.
